# Treatment of a subdural empyema complicated by intracerebral abscess due to *Brucella* infection

**DOI:** 10.1590/1414-431X20165712

**Published:** 2017-03-30

**Authors:** J. Zhang, Z. Chen, L. Xie, C. Zhao, H. Zhao, C. Fu, G. Chen, Z. Hao, L. Wang, W. Li

**Affiliations:** 1 Department of Neurosurgery, China-Japan Union Hospital, Jilin University, Changchun, Jilin Province, China; 2 Department of Vascular Surgery, China-Japan Union Hospital, Jilin University, Changchun, Jilin Province, China; 3 Department of Pathology, China-Japan Union Hospital, Jilin University, Changchun, Jilin Province, China; 4 Department of Clinical Laboratory, China-Japan Union Hospital, Jilin University, Changchun, Jilin Province, China

**Keywords:** Subdural empyema, Neurobrucellosis, Craniotomy, Infection, Treatment

## Abstract

A 55-year-old male presented with fever, stupor, aphasia, and left hemiparesis. A history of head trauma 3 months before was also reported. Cranial magnetic resonance imaging revealed slight contrast enhancement of lesions under the right frontal skull plate and right frontal lobe. Because of deterioration in nutritional status and intracranial hypertension, the patient was prepared for burr hole surgery. A subdural empyema (SDE) recurred after simple drainage. After detection of *Brucella* species in SDE, craniotomy combined with antibiotic treatment was undertaken. The patient received antibiotic therapy for 6 months (two doses of 2 g ceftriaxone, two doses of 100 mg doxycycline, and 700 mg rifapentine for 6 months) that resulted in complete cure of the infection. Thus, it was speculated that the preexisting subdural hematoma was formed after head trauma, which was followed by a hematogenous infection caused by *Brucella* species.

## Introduction

Brucellosis is an infectious disease caused by bacteria from *Brucella* species. Brucellosis involving the central nervous system (CNS) is termed neurobrucellosis. The most common complications are meningitis and meningoencephalitis. Only 2.7% of meningitis cases develop into a subdural abscess (subdural empyema, SDE) (1). Only one case of SDE caused by *Brucella* species has been reported. Herein, we report for the first time the treatment of an SDE complicated by an intracerebral abscess due to an infection caused by *Brucella* species. A craniotomy revealed thick yellowish purulent material in SDE, and a large number of red blood cells as well as inflammatory cells were observed under the microscope. Thus, we presumed that the preexisting subdural hematoma, aggravated after head trauma, was followed by hematogenous infection by *Brucella* species. The patient was cured with medication and surgery.

## Case Report

A 55-year-old male presented fever, stupor, aphasia, and left hemiparesis for 1 month. Two months before, when the patient suffered mild brain injury and had occasional headaches, aphasia and limb weakness were not present. There was no history of hypertension, heart disease, nasosinusitis, tympanitis, mastoiditis, or craniocerebral surgery. Three months earlier, the patient had a direct contact with diseased animals in a Brucellosis epidemic region (consuming unpasteurized milk products). Upon hospital admission, vital signs were as follows: body temperature 38.5°C, pulse 85 bpm, respiratory rate 19/min, and blood pressure 123/75 mmHg.

**Figure 1:**
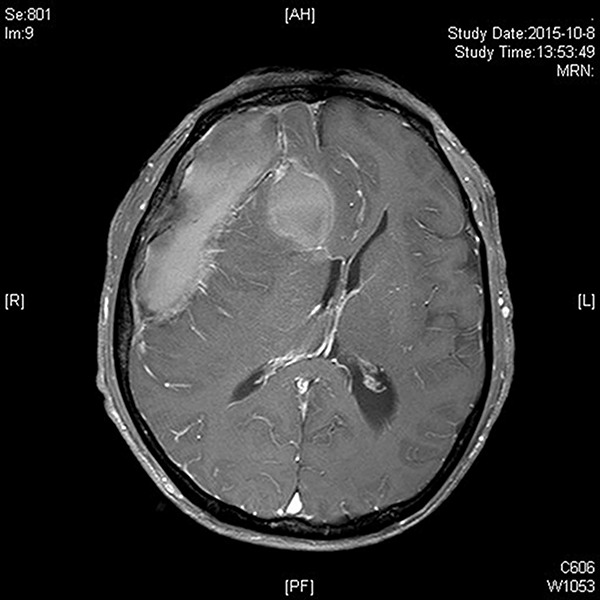
Admission magnetic resonance imaging of the patient.

Magnetic resonance imaging (MRI) of the cranium revealed a lesion with fusiform annular contrast enhancement under the right frontal cranium, and a lesion with circular annular contrast enhancement in the right frontal lobe with the distinct shifting of median structures (Figure 1). Routine blood analyses showed white blood cell count at 6.85×10^9^/L, neutrophils at 57.2%, and lymphocytes at 34.4%. A deficient nutritional status was also present.

Surgery aiming to relieve intracranial pressure (ICP) was planned. Drainage was carried out and pus removed. The abscess in the frontal lobe was treated by antibiotic therapy (vancomycin plus meropenem) due to its small size. Cultures of pus and blood were negative for bacterial infection. Upon lumbar puncture, cerebrospinal fluid (CSF) pressure was normal, and fluid was turbid. Test results revealed CSF white blood count of 146×10^6^/L, protein level of 3 g/L, and glucose level of 2.0 mmol/L.

**Figure 2:**
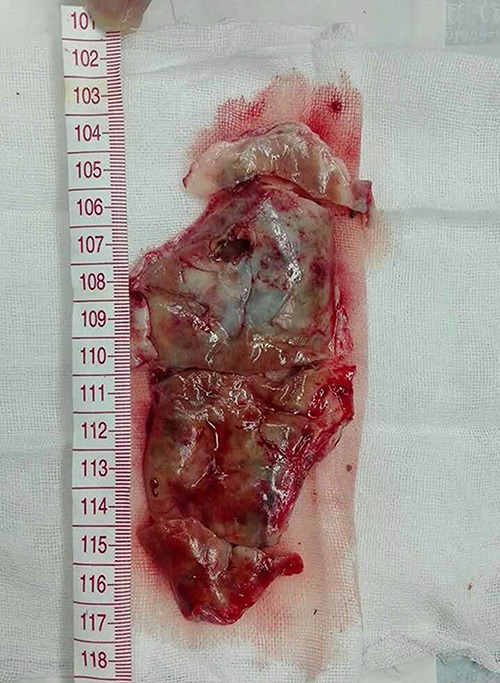
Pathological specimen of the abscess excised by craniotomy.

**Figure 3:**
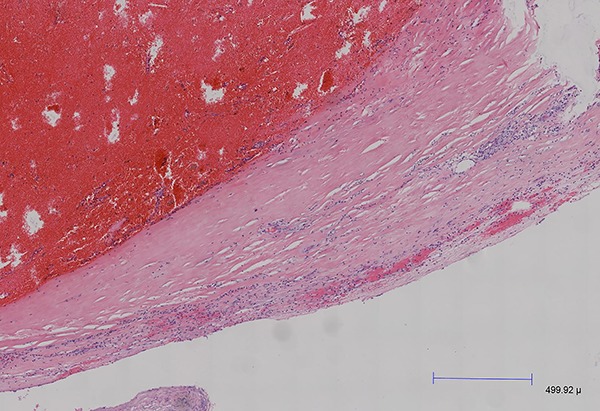
Photomicrograph of the excised abscess wall.

One week later, cranial MRI revealed a clear increase of the SDE and the abscess in the frontal lobe had not improved. SDE excision was carried out by craniotomy and an abscess (14×4 cm) between the dura mater and arachnoid mater was excised. The abscess was yellow, rigid, and filled with a necrotic material (Figure 2). After SDE removal, the abscess in the frontal lobe was removed by ultrasound. There was no communication between the two nidi. Blood culture of SDE revealed *Brucella* species. The serum agglutination test (SAT) for *Brucella* species was carried out in the Changchun Prefecture Center for Disease Prevention and Control (Changchun, China); the titer was 1:100, and hence, infection by *Brucella* species was confirmed. The patient was treated with two doses of 2 g ceftriaxone, two doses of 100 mg doxycycline, and 700 mg rifapentine for 6 months. We observed a significant number of red blood cells and inflammatory cells in the abscess under the microscope (Figure 3).

Six months later, the patient did not present fever, limbs were stronger than before, and he could live independently. Follow-up cranial MRI showed shrinkage of nidi and weakening of contrast enhancement. Also, the agglutination test results were negative.

## Discussion

*Brucella* species are small, Gram-negative, non-motile, non-spore-forming, rod-shaped bacteria, and are involved in systemic diseases (e.g., digestive tract, urogenital system, nervous system). Brucellosis is prevalent in the Mediterranean basin, India, Arabian Peninsula, Mexico, and South America. The Nei Mongol Autonomous Region, Jilin Province, Heilongjiang Province, and Xinjiang Uygur Autonomous Region have documented evidence of individuals consuming unpasteurized milk or milk products and residing in close contact with animals and their products. In the current case, the patient resided in an area with a high incidence of brucellosis, and also presented a history of consuming raw milk. Thus, we speculated that the infection occurred by the oral port. Brucellosis is a treatable disease with a favorable outcome; however, patients may be either not diagnosed or not treated rapidly. Complications are often severe and life-threatening. Neurobrucellosis is a rare complication of brucellosis and often overlooked due to its non-specific manifestations and a broad spectrum of syndromes with similar characteristics (2). In 2008, the number of cases of brucellosis reported worldwide was >500,000, and 1.7 to 10% of patients with neurobrucellosis suffer morbidity (3).

*Brucella* species can impact the nervous system through allergic reactions, an immune response, or apoptosis. Meningitis is a common consequence of neurobrucellosis. Other neurologic complications include meningoencephalitis, myelitis-radiculoneuritis, brain abscess, and epidural abscess; SDE is rare. Brain abscesses due to brucellosis at various locations are well-known entities. SDE complicated by an intracerebral abscess caused by infection of *Brucella* species has not yet been reported. Only Shoshan et al. (4) reported a case of SDE caused by *Brucella* species infection in 1996. SDE can develop after otitis media, sinusitis, mastoiditis, meningitis, trauma, and after craniotomy. Few reported cases were associated with minor head trauma (5). Common pathogens of SDE are *Streptococcus pneumoniae* and meningococci. *S. aureus* infection can commonly occurr post-traumatically (6). SDE develops as a complication of bacterial meningitis resulting in significant morbidity and mortality, despite recent advances in neuroimaging, surgical techniques, and antibiotic therapy (7).

The diagnostic certainty of neurobrucellosis requires isolation of the organism from the CSF; however, this is not a simple process. A serological diagnosis can be made with the Coombs test on CSF. In the case of our patient, the CSF examination was not suitable because the cranial MRI showed high ICP. The diagnosis of neurobrucellosis requires a SAT titer >1:160 or any positive titer in CSF. The sensitivity of the cerebrospinal agglutination test (CSFAT) was higher than that of the SAT. This is because *Brucella* antibodies cannot be detected in CSF samples from patients who have systemic brucellosis without neurologic involvement, rather only in CSF samples from patients with proven neurobrucellosis. Haji-Abdolbagi et al. reported 16 cases of brucellosis. Their CSFAT titer was >1/160 in 14 cases (87.5%), and the SAT titer was >1/160 in 3 cases (10%) (8). Bodur et al. (9) studied 13 cases: CASAT revealed 12 cases to be positive compared with the blood culture (3 positive cultures) and CSF culture (3 positive cultures). The gold standard for the diagnosis is a positive result in the blood or CSF culture. However, *Brucella* species grow slowly *in vitro* and positivity can be <50%, hence, rapid identification of pathogens can be very difficult. Our results are in accordance with the reports stating that the culture of *Brucella* species is frequently negative, the prevalence of positive results is low, and the period of culture is long.

If brucellosis is strongly suspected and the CSFAT is negative, then the CSF Coombs agglutination test is more sensitive (10). In the current patient, the SAT was positive, the culture of the abscess was negative, and lumbar puncture was not performed because of high ICP. Hence, we could not carry out the CSFAT at an early stage. Brucellosis is often missed because of its various symptoms and negative laboratory results. Initially, our patient was not considered to have brucellosis, which indicates that this disease requires attention in endemic areas in order to facilitate an early diagnosis and timely treatment.

SDE can be a fatal neurosurgical emergency that may evolve rapidly and cause cerebral hernia. Identification of the SDE and subdural hematoma using computed tomography is difficult. MRI has higher sensitivity for SDE identification. Interestingly, the disease course in our patient was chronic. The capsule formed gradually in the SDE and the lesions due to *Brucella* infection developed slowly. Thus, we speculated that preexisting subdural hematoma had formed after minor head trauma, and hematogenous infection by *Brucella* species had occurred via the outer membrane resulting in empyema. Limura et al. (11) reported a patient with infected subdural hemorrhage.

A multicenter retrospective study in 2011 showed that the most adequate treatment protocol for brucellosis is comprised of ceftriaxone (two doses of 2 g), rifampin (600–900 mg), and doxycycline (two doses of 100 mg). The treatment period was significantly shorter (median = 5 months) than an oral treatment protocol (median = 6 months) (12). However, the surgical procedure for SDE is controversial. The literature suggests that a single intracerebral abscess caused by bacillus can be cured by antibiotics alone. Kizilkilic et al. (13) used the antibiotic treatment solely to cure a case of brucellosis in the cerebellum. However, for our patient the lesion was a SDE and the medication was not effective. Munusamy et al. (14) suggested that if the pus is relatively thin and a capsule has not formed, then drilling to drain the SDE is feasible, thereby avoiding the severe complications associated with craniotomy to clear the SDE. If the SDE is more viscous and developed, craniotomy would be necessary, and simple drainage by drilling may lead to SDE recurrence. If the brain swells, then bone-flap decompression can be carried out. Inoue et al. (15) arrived at a similar conclusion, stating that craniotomy can remove the SDE, and that “rinsing” the abscess cavity thoroughly is critical.

In the present patient, drainage was selected initially as it is a safe and easy approach for the subdural space. Drainage of the abscess in the frontal lobe was planned by stereotactic puncture but the SDE recurred. One week later, computed tomography showed SDE recurrence; thus, we carried out a craniotomy. The reason for SDE recurrence was the thickness and rigidity of the SDE wall. Hence, simple drainage was not curative. When the culture results showed *Brucella* species, we switched the antibiotics to ceftriaxone, rifapentine, and doxycycline. Hence, if we had opted for craniotomy at the beginning, the SDE probably would not have recurred.

Immediate craniotomy and use of appropriate anti-*Brucella* agents would have treated this rare case of SDE, complicated by an intracerebral frontal abscess due to infection by *Brucella* species and severe SDE complicated by an intracerebral abscess. This management plan should be implemented in similar cases.
